# Unravelling the effects of mechanical physiological conditioning on cardiac adipose tissue-derived progenitor cells *in vitro* and *in silico*

**DOI:** 10.1038/s41598-017-18799-5

**Published:** 2018-01-11

**Authors:** Aida Llucià-Valldeperas, Ramon Bragós, Carolina Soler-Botija, Santiago Roura, Carolina Gálvez-Montón, Cristina Prat-Vidal, Isaac Perea-Gil, Antoni Bayes-Genis

**Affiliations:** 1 ICREC Research Program, Health Science Research Institute Germans Trias i Pujol, Badalona, Spain; 2 Electronic and Biomedical Instrumentation Group, Departament d’Enginyeria Electrònica, Universitat Politècnica de Catalunya, Barcelona, Spain; 3 Center of Regenerative Medicine in Barcelona, Barcelona, Spain; 4 Cardiology Service, Germans Trias i Pujol University Hospital, Badalona, Spain; 5 Department of Medicine, Universitat Autònoma de Barcelona, Barcelona, Spain; 6 CIBER Cardiovascular, Instituto de Salud Carlos III, Madrid, Spain

## Abstract

Mechanical conditioning is incompletely characterized for stimulating therapeutic cells within the physiological range. We sought to unravel the mechanism of action underlying mechanical conditioning of adipose tissue-derived progenitor cells (ATDPCs), both *in vitro* and *in silico*. Cardiac ATDPCs, grown on 3 different patterned surfaces, were mechanically stretched for 7 days at 1 Hz. A custom-designed, magnet-based, mechanical stimulator device was developed to apply ~10% mechanical stretching to monolayer cell cultures. Gene and protein analyses were performed for each cell type and condition. Cell supernatants were also collected to analyze secreted proteins and construct an artificial neural network. Gene and protein modulations were different for each surface pattern. After mechanostimulation, cardiac ATDPCs increased the expression of structural genes and there was a rising trend on cardiac transcription factors. Finally, secretome analyses revealed upregulation of proteins associated with both myocardial infarction and cardiac regeneration, such as regulators of the immune response, angiogenesis or cell adhesion. To conclude, mechanical conditioning of cardiac ATDPCs enhanced the expression of early and late cardiac genes *in vitro*. Additionally, *in silico* analyses of secreted proteins showed that mechanical stimulation of cardiac ATDPCs was highly associated with myocardial infarction and repair.

## Introduction

The heart is a mechanically-active organ that dynamically senses and responds according to its local environmental milieu. This environment fluctuates on a beat-to-beat basis, with additional modulation from respiratory activity, postural changes and physical activity; moreover, this milieu is further enriched by physiological (e.g., pregnancy, endurance training) and pathological (e.g., pressure or volume overload) conditions^1^. The heart is not a simple pump; it detects changes in mechanical demands and adjusts its performance via changes in heart rate and stroke volume^2^. These regulatory processes are encoded and maintained intramyocardially. However, the mechanisms underlying cardiac mechanosensitivity are poorly understood^3^.

It is well known that mechanical stretch improves contractility^4^, enables growth factor secretion, and promotes calcium handling in cardiac myocytes. Additionally, mechanical stretch alters extracellular matrix (ECM) synthesis in cardiac fibroblasts^5^. Generally, mechanical tension promotes heart muscle survival by regulating cell alignment, elongation, hypertrophy, and differentiation^6^.

In contractile tissues, functional properties are directly related to cellular orientation and elongation. Thus, cell function critically depends on the interaction between multiple guidance cues, such as topography^7^. On the other hand, the connective tissue provides passive support for regulating heart tensile strength and stiffness. Indeed, its endomysial part provides support for heart compliance and protection against overstretch thanks to its laminae organization^8^.

Nevertheless, there is no evidence on mechanical conditioning effects on adipose tissue-derived progenitor cells (ATDPCs), which are currently gaining momentum for clinical translation in the context of a variety of tissue regeneration trials such as APOLLO (NCT00442806)^9^, ADVANCE (NCT01216995), and PRECISE (NCT00426868)^10^ for myocardial infarction (MI) repair. In particular, ATDPCs are reported to exercise beneficial effects through two mechanisms: cell differentiation and paracrine signaling^11^. Indeed, ATDPCs-derived secretome has been associated with angiogenesis, immunomodulation, and wound healing^12^. In the same line, proteome analysis can generate Artificial Neural Networks (ANN), which evaluate possible relations among proteins sets inside the network and quantify this probability.

This study aimed to provide a better understanding of the effects of mechanical conditioning on cardiac ATDPCs. To that end, we designed and constructed a magnet-based, mechanical stimulator device to apply ~10% mechanical stretching to *in vitro* cultures of cell monolayers. We then cultured ATDPCs on 3 different patterned surfaces and continuously applied mechanical stretching to explore their gene expression and secretome. Finally, based on that data, we constructed and analyzed an ANN *in silico*.

## Results

### Mechanical Stimulation Validation

**Figure 1:**
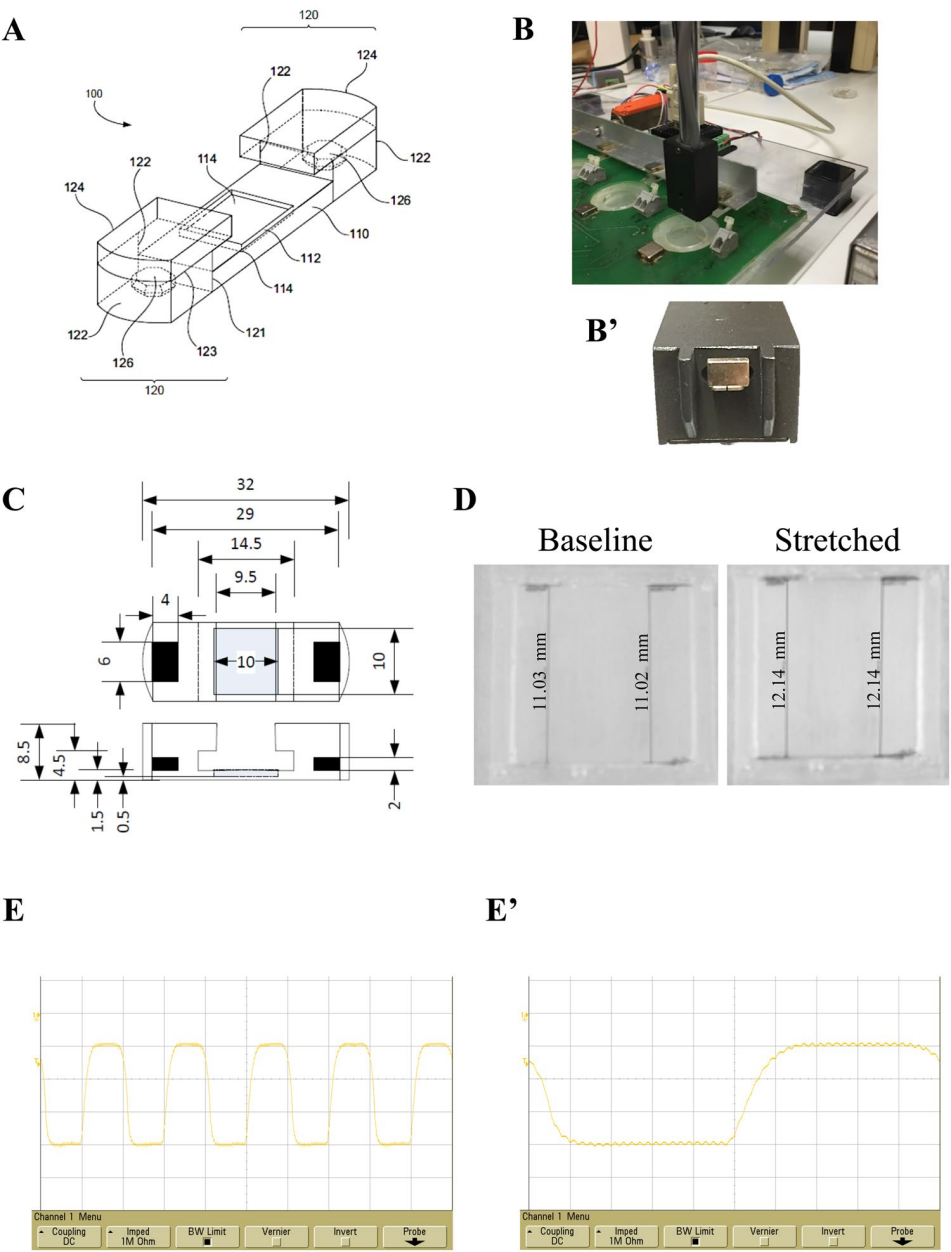
Mechanical stimulator design, characterization, and validation. (**A**) 3D view of the cell support system as reported in the patent PCT/EP 2012/061224. Two lateral structures, each containing an embedded magnet (part no. 126), are designed to exert pulling force on a thinner, central segment, which contains a rectangular cavity (pool, part no. 114) that holds a cell culture solution for growing the cell monolayer. (**B**) The Fort25 force transducer is shown, with a magnet attached to the sensor and aligned with the moving magnet; (**B′**) detail of the magnet attached to the force sensor lever. (**C**) Dimensions (in mm) of the cell support system as reported in the patent PCT/EP 2012/061224. The central segment containing the pool is the thinnest section of the whole structure; thus, it undergoes the greatest deformation. (**D**) Longitudinal and transverse measurements (in pixels) of the inner borders of the pool shows a ~10% elongation of the longitudinal borders. (**E**) Capture of the output differential voltage detected with the Fort25 sensor. (**E′**) The rise time slope is shown on a magnified scale. The rise and fall times were set to 100 ms, for a rough imitation of the ventricular pressure waveform. The small oscillations represent detection of residual, 50 Hz interference from the mains voltage supply (background noise).

The dimensions of the final structure (Fig. 1A) are specified in Fig. 1C (in mm). The pool that held the cell monolayer had a surface area of 1 × 1 cm and a depth of 1 mm. The bottom of the pool was a transparent film of 0.5 mm that allowed microscopic observation and the imprint of a pattern. The PDMS had been cured at room temperature and had a Young’s modulus of 1.3 MPa^13^, close to physiological levels^14^. Considering these parameters, a force of 0.104 N (10.6 gf) was required to provide a strain of 1%, and a force of 1.04 N (106 gf) provided a strain of ~10%.

Strain was measured with the Matlab (Mathworks) ImgTool utility display. When the moving magnet was positioned next to the silicone construct, both sides of the pool underwent a longitudinal deformation of ~10%, and the transverse borders of the pool underwent negligible deformation (Fig. 1D). The stretch was transferred from the substrate to the cell monolayer, as evidenced by the microscale strain transfer characterization (data not shown). Moreover, the Fort25 sensor (Fig. 1B) detected a trapezoidal waveform with a rise and fall times of 100 ms, mimicking the pressure cycle shape in the heart (Fig. 1E,E′).

### Gene expression analysis

After 7 days of mechanical conditioning, cells grown on different patterned surfaces (vertical, horizontal, or smooth) were harvested, and RNA was isolated for gene expression experiments. We found that mechanical stretching modulated gene expression in both cardiac and subcutaneous ATDPCs. Moreover, the modulations were strongly dependent on the surface pattern (Table 1 and Supplementary Table 1). After mechanical stimulation, cardiac ATDPCs significantly increased expression of structural genes and evidenced a rising trend in cardiac transcription factors (Table 1). Structural genes, such as cardiac troponin I (cTnI) and α-actinin, were significantly upregulated in cells grown on horizontal (*P* = 0.044) and smooth (*P* = 0.001) surfaces, respectively. Additionally, there was an augmenting trend for GATA-4 of ∼3-fold in cells grown on vertical patterned surfaces (*P* = 0.068), and Tbx5 of ∼1.4-fold in cells grown on horizontal patterned surfaces (*P* = 0.065). On the other hand, in subcutaneous ATDPCs, mechanostimulation augmented Tbx5 gene expression ∼2-fold on vertical patterned surfaces (*P* = 0.007), and SERCA2 expression presents an increasing tendency of ∼1.5-fold on horizontal patterned surfaces (*P* = 0.079) (Supplementary Table 1).

**Table 1:** Relative expression of cardiac markers for each surface condition in cardiac ATDPC cultures.

	Sample	Tbx5	MEF2A	GATA-4	α-actinin	Cx43	SERCA2	β-MyHC	cTnI
Vertical	cardiac ATDPCs Con	0.003 ± 0.001	0.089 ± 0.018	0.428 ± 0.196	2.445 ± 1.089	0.705 ± 0.253	0.397 ± 0.237	0.095 ± 0.049	0.693 ± 0.555
	cardiac ATDPCs MS	0.004 ± 0.002	0.169 ± 0.089	1.209 ± 0.378	2.326 ± 1.109	1.371 ± 0.949	0.551 ± 0.413	0.181 ± 0.114	0.964 ± 0.620
	Ratio MS/Con	**1.430**	**1.901**	**2.822**	**0.951**	**1.945**	**1.386**	**1.912**	**1.391**
	*P*-value Con vs MS	0.653	0.410	**#0.068**	0.753	0.517	0.756	0.443	0.940
Horizontal	cardiac ATDPCs Con	0.004 ± 0.000	0.031 ± 0.007	0.030 ± 0.005	0.180 ± 0.011	0.150 ± 0.043	0.046 ± 0.007	7.8·10^−6^ ± 5.4·10^−6^	5.1·10^−6^ ± 2·10^−6^
	cardiac ATDPCs MS	0.005 ± 0.000	0.033 ± 0.002	0.035 ± 0.003	0.286 ± 0.064	0.139 ± 0.042	0.063 ± 0.005	1.8·10^−5^ ± 1.5·10^−5^	1.4·10^−5^ ± 1.9·10^−6^
	Ratio MS/Con	**1.405**	**1.067**	**1.179**	**1.587**	**0.926**	**1.384**	**2.320**	**2.672**
	*P*-value Con vs MS	**#0.065**	0.854	0.397	0.181	0.865	0.110	0.419	*0.044
Smooth	cardiac ATDPCs Con	0.001 ± 0.000	0.029 ± 0.007	0.050 ± 0.008	0.213 ± 0.010	0.228 ± 0.100	0.091 ± 0.015	1.7·10^−5^ ± 1.6·10^−5^	7.6·10^−6^ ± 5.1·10^−6^
	cardiac ATDPCs MS	0.001 ± 0.000	0.045 ± 0.014	0.067 ± 0.013	0.362 ± 0.018	0.262 ± 0.101	0.127 ± 0.019	2·10^−5^ ± 1.5·10^−5^	1.2·10^−5^ ± 1.2·10^−5^
	Ratio MS/Con	**1.716**	**1.537**	**1.326**	**0.891**	**1.151**	**1.390**	**1.182**	**1.547**
	*P*-value Con vs MS	0.217	0.404	0.312	*0.001	0.817	0.198	0.848	0.662

Gene expressions were analyzed in duplicate for comparisons between mechanically stimulated (MS) and control (Con) samples. Relative expression (2^−ΔCT^) and fold-changes in expression (MS/Con) are shown for cardiomyogenic genes. Values were normalized to GAPDH expression and represent the mean ± SEM for at least 4 independent experiments. *P < 0.05 (significant) and ^#^*P* < 0.10 (trend).

### Protein expression analysis

**Figure 2:**
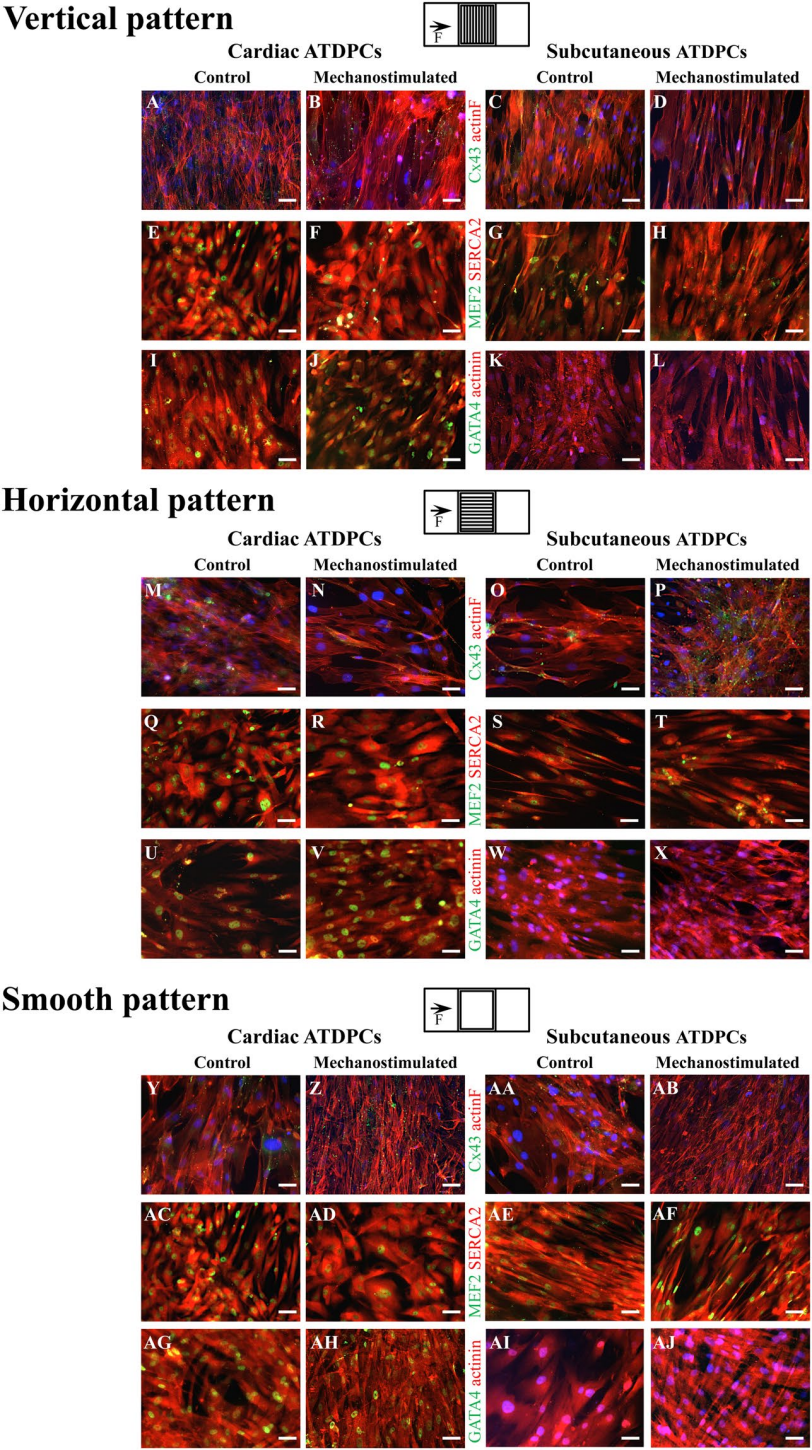
Protein expression profiles after mechanical stimulation. Immunohistochemical staining shows expression of proteins in cardiac (left) and subcutaneous (right) ATDPCs grown on vertical, horizontal, and smooth patterned surfaces, as indicated (diagrams in the top, centers indicate surface patterns with respect to the magnets in the cell support system). Control (first and third columns) and mechanostimulated (second and fourth columns) were stained, as indicated, to show expression of (top rows) actin F (phalloidin staining, red) and Cx43 (green); (center rows) SERCA2 (red) and MEF2 (green); and (bottom rows) sarcomeric α-actinin (red) and GATA-4 (green). Nuclei were counterstained with DAPI (blue) in **A**–**D**,**K**,**L**,**M**–**P**,**W**,**X**,**Y**–**AB**,**AI**,**AJ**). Scale bars = 50 µm.

Gene modulations observed were also translated at the protein level; protein expression profiles of the main cardiac markers in non-conditioned controls and mechanically-stimulated ATDPCs grown on each patterned surface are shown in Fig. 2. In both cell lines and in all patterns and conditions, Cx43 was distributed throughout the cytoplasm, but particularly at the plasma membrane and at the cell poles (Fig. 2 upper rows). SERCA2 and α-actinin were observed in the cytoplasm of cells but without an appreciable mature sarcomere organization (Fig. 2 middle and lower rows, respectively). MEF2A and GATA-4 are nuclear markers (Fig. 2 middle and lower rows, respectively); and GATA-4 protein expression was detected only in the nuclei of cardiac ATDPCs, not in subcutaneous ATDPCs (Fig. 2 lower rows).

Cell monolayers showed some degree of alignment with the different surface patterns; while cells were randomly distributed on smooth surfaces, mainly dictated by cell density. Fluorescence intensities were normalized and measured to quantify protein abundance in each condition. Protein increments observed at gene level were revealed and corroborated at protein level (Table 2).

**Table 2:** Protein quantification determined from immunostaining of the main cardiac markers for each condition and cell type.

	Sample	MEF2A	GATA-4	α-actinin	Cx43	SERCA2	actinF
Vertical	cardiac ATDPCs Con	1.125 ± 0.093	0.979 ± 0.114	0.958 ± 0.088	0.449 ± 0.071	1.325 ± 0.110	0.649 ± 0.069
	cardiac ATDPCs MS	1.289 ± 0.179	0.729 ± 0.016	0.705 ± 0.024	0.678 ± 0.028	2.065 ± 0.184	0.729 ± 0.019
	Ratio MS/Con	**1.146**	**0.745**	**0.737**	**1.509**	**1.559**	**1.123**
	*P*-value Con vs MS	0.423	*0.050	*0.016	*0.002	*0.002	0.301
Horizontal	cardiac ATDPCs Con	1.150 ± 0.146	0.654 ± 0.024	1.113 ± 0.014	0.448 ± 0.117	1.213 ± 0.054	1.233 ± 0.270
	cardiac ATDPCs MS	0.732 ± 0.071	0.662 ± 0.020	1.499 ± 0.051	1.080 ± 0.020	1.521 ± 0.080	1.425 ± 0.044
	Ratio MS/Con	**0.636**	**1.013**	**1.346**	**2.409**	**1.254**	**1.156**
	*P*-value Con vs MS	*0.011	0.880	*0.000	*0.000	*0.043	0.504
Smooth	cardiac ATDPCs Con	0.435 ± 0.012	0.725 ± 0.070	1.126 ± 0.086	0.858 ± 0.068	0.435 ± 0.017	1.330 ± 0.095
	cardiac ATDPCs MS	0.639 ± 0.015	0.910 ± 0.088	2.568 ± 0.215	1.794 ± 0.160	0.975 ± 0.027	5.190 ± 0.617
	Ratio MS/Con	**1.469**	**1.255**	**2.281**	**2.091**	**2.242**	**3.904**
	*P*-value Con vs MS	*0.000	0.123	*0.000	*0.000	*0.000	*0.000
Vertical	subcutaneous ATDPCs Con	0.841 ± 0.047	NA	1.403 ± 0.169	0.776 ± 0.147	0.613 ± 0.048	0.751 ± 0.108
	subcutaneous ATDPCs MS	0.784 ± 0.096	NA	1.535 ± 0.367	1.048 ± 0.110	0.508 ± 0.043	1.677 ± 0.123
	Ratio MS/Con	**0.932**		**1.094**	**1.349**	**0.828**	**2.232**
	*P*-value Con vs MS	0.562		0.726	0.197	0.110	*0.000
Horizontal	subcutaneous ATDPCs Con	0.718 ± 0.058	NA	1.223 ± 0.043	0.972 ± 0.013	1.572 ± 0.047	1.287 ± 0.095
	subcutaneous ATDPCs MS	0.711 ± 0.017	NA	1.172 ± 0.043	1.005 ± 0.037	1.178 ± 0.059	1.406 ± 0.057
	Ratio MS/Con	**0.990**		**0.959**	**1.034**	**0.749**	**1.093**
	*P*-value Con vs MS	0.891		0.492	0.405	*0.000	0.264
Smooth	subcutaneous ATDPCs Con	0.662 ± 0.048	NA	1.194 ± 0.099	0.752 ± 0.095	1.542 ± 0.366	1.955 ± 0.254
	subcutaneous ATDPCs MS	0.965 ± 0.063	NA	1.121 ± 0.080	1.520 ± 0.112	2.377 ± 0.158	2.823 ± 0.229
	Ratio MS/Con	**1.457**		**0.938**	**2.022**	**1.542**	**1.445**
	*P*-value Con vs MS	*0.004		0.568	*0.000	**#0.052**	*0.018

Fluorescence intensity values were normalized to DAPI intensity and are shown as the mean ± SEM. Con: control, MS: mechanostimulated. *P < 0.05 (significant) and ^#^*P* < 0.10 (trend). NA, not applicable.

### Secretome analysis

**Figure 3:**
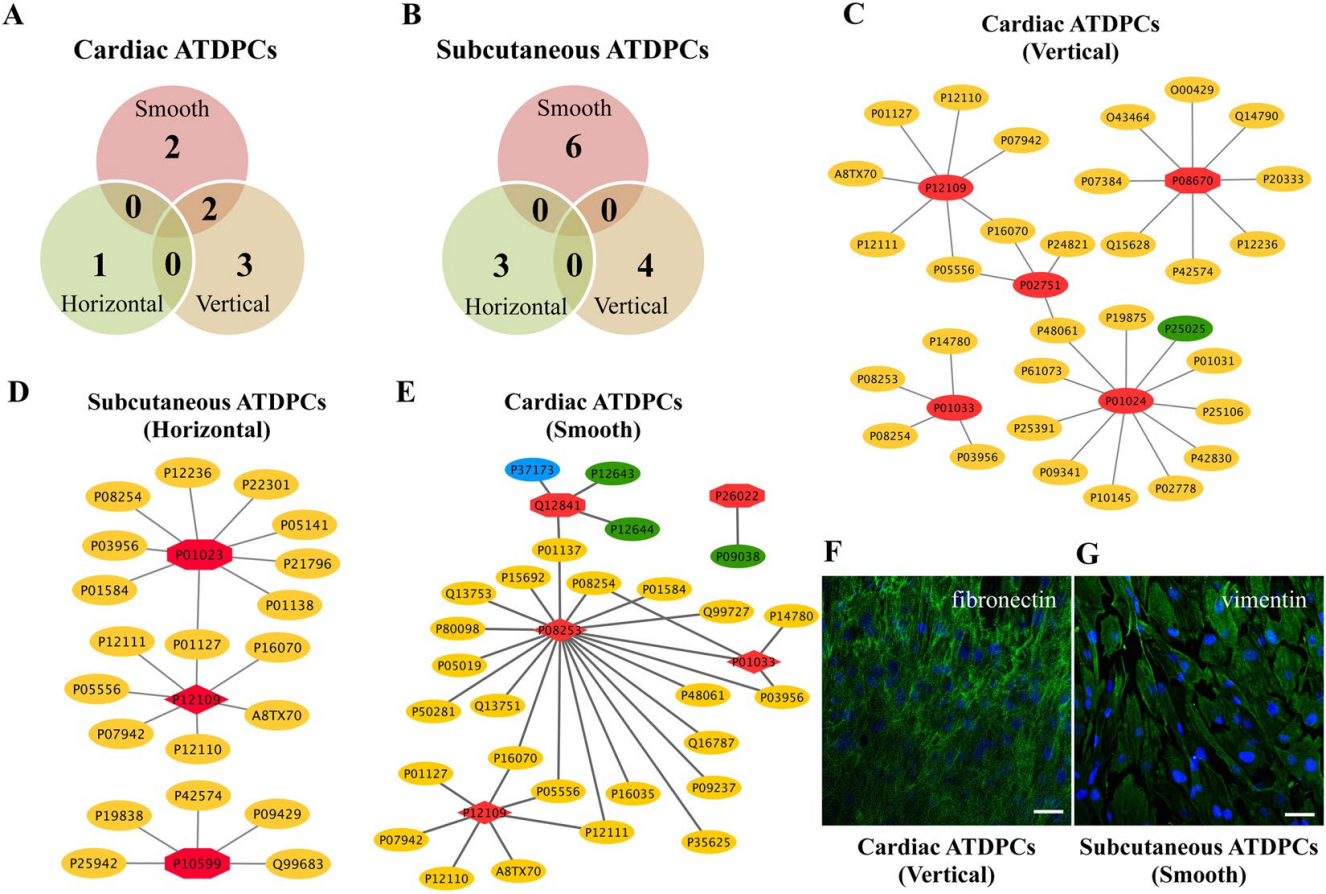
Secretome analysis. (**A**,**B**) Venn’s diagrams of differentially expressed proteins in (**A**) cardiac ATDPCs and (**B**) subcutaneous ATDPCs. (**C–E**) Protein networks for (**C)** cardiac seeded on vertical patterned surfaces, (**D**) subcutaneous ATDPCs seeded on horizontal patterned surfaces, and (**E**) cardiac ATDPCs grown on a smooth surface. Red octagons are secretome proteins; red diamonds are secretome proteins that are also effectors of MI or cardiac regeneration; yellow circles are effectors of MI or cardiac regeneration that are linked to the secretome proteins; the blue circle indicates a protein included in the Gene Ontology (GO) terms that was linked to the indicated secretome protein; and green circles indicate proteins or effectors linked to the secretome proteins. (**F**) Fibronectin protein expression (green) in mechanically stimulated cardiac ATDPCs grown on vertical patterned surfaces. (**G**) Vimentin protein expression (green) in mechanically stimulated subcutaneous ATDPCs grown on smooth surfaces. Nuclei were counterstained with DAPI (blue). Scale bars = 50 µm.

About 200 proteins were identified in the supernatants collected from control and mechanically-stimulated cardiac and subcutaneous ATDPCs on each patterned surface. Statistical analyses revealed differential expression of several proteins (Table 3), and some proteins were identified in different secretomes (Fig. 3A,B).

**Table 3:** Results from the pairwise, trimmed, mean normalization analysis for each condition.

Experiment	Protein name	UNIPROT	MS *vs* Con	Effector	# Linked Effectors
Cardiac ATDPCs vertical	Complement C3	P01024	1.017	Inflammation	11
	Tissue inhibitor of metalloproteinases 1 (TIMP-1)	P01033	0.911	LV ECM remodelling	4
	Fibronectin (FN)	P02751	1.004	LV ECM remodelling	4
	Vimentin	P08670	0.974	Intermediary	8
	Collagen type VI α-1 chain	P12109	1.169	LV ECM remodelling	7
Cardiac ATDPCs horizontal	Stromal interaction molecule 2	Q9P246	*de novo*	—	0
Cardiac ATDPCs smooth	Tissue inhibitor of metalloproteinases 1 (TIMP-1)	P01033	1.002	LV ECM remodelling	4
	Matrix metalloproteinase 2 (MMP-2)	P08253	1.036	LV ECM remodelling	20
	Collagen type VI α-1 chain	P12109	1.097	LV ECM remodelling	7
	Pentraxin-related protein 3 (PTX3)	P26022	1.166	Intermediary	1
	Follistatin-related protein 1 (FSTL1)	Q12841	0.994	Proliferation	4
Subcutaneous ATDPCs vertical	Tissue inhibitor of metalloproteinases 1 (TIMP-1)	P01033	1.063	LV ECM remodelling	4
	Fibronectin (FN)	P02751	1.012	LV ECM remodelling	4
	Matrix metalloproteinase 2 (MMP-2)	P08253	0.964	LV ECM remodelling	20
	Stromal interaction molecule 2	Q9P246	1.028	—	0
Subcutaneous ATDPCs horizontal	α-2-macroglobulin (α-2-M)	P01023	1.008	Intermediary	10
	Thioredoxin (TXN)	P10599	1.038	Intermediary	5
	Collagen type VI α-1 chain	P12109	0.824	LV ECM remodelling	7
Subcutaneous ATDPCs smooth	Collagen type I α-1 chain	P02452	1.031	Intermediary	7
	Collagen type I α-2 chain	P08123	1.017	Intermediary	5
	Vimentin	P08670	1.006	Intermediary	8
	Complement C1s subcomponent	P09871	1.089	—	0
	Transforming growth factor-β-induced protein ig-h3	Q15582	0.993	—	0
	Nuclear pore complex protein (Nup205)	Q92621	1.009	Intermediary	2

Proteins that showed significant differential expression in mechanostimulated (MS) and control (Con) cells are shown with their name and UNIPROT code. The relative protein abundance is shown in the “MS *vs*. Con” column. The “Effector” column indicates the protein category. The “# Linked Effectors” column indicates the number of effector proteins linked to the protein of interest.

The ANN analysis (Table 4) showed that most secretomes were consistently associated with MI (~80% predictability). Secretomes from cardiac ATDPCs seeded on smooth surfaces and subcutaneous ATDPCs seeded on horizontal surfaces were also associated with cardiac regeneration (~70–80% predictability) and its two sub-functions, angiogenesis and proliferation. Further, stimulated cardiac and subcutaneous ATDPCs grown on vertical and horizontal surfaces, respectively, were more related to MI and its two sub-functions, inflammation and left ventricle (LV) ECM remodeling, than ATDPCs grown in the other conditions. The 3 scenarios most closely related to MI and cardiac regeneration were cardiac ATDPCs mechanically stimulated on vertical and smooth surfaces, and subcutaneous ATDPCs mechanically stimulated on horizontal surfaces. In these 3 conditions, secreted proteins were primarily involved in ECM remodeling, inflammation, and proliferation (Table 3, Fig. 3C–E).

**Table 4:** Artificial Neural Network prediction values for myocardial infarction (MI) and cardiac regeneration functions.

Treatment	Myocardial Infarction				
	MI	MI Inflammation	Cardiomyocyte cell death	LV ECM remodelling	
Cardiac ATDPCs vertical	**73.28%**	**58.38%**	**21.04%**	**84.30%**	
Cardiac ATDPCs horizontal	4.96%	13.20%	57.91%	21.92%	
Cardiac ATDPCs smooth	79.07%	49.59%	11.96%	88.26%	
Subcutaneous ATDPCs vertical	81.84%	32.05%	5.24%	82.73%	
Subcutaneous ATDPCs horizontal	**74.34%**	**70.17%**	**29.99%**	**71.01%**	
Subcutaneous ATDPCs smooth	38.13%	51.36%	39.83%	29.14%	
**Treatment**	**Cardiac Regeneration**				
	**Cardiac Regeneration**	**Angiogenesis**	**Cell survival signalling**	**Differentiation**	**Proliferation**
Cardiac ATDPCs vertical	15.94%	26.80%	5.32%	14.04%	5.39%
Cardiac ATDPCs horizontal	5.29%	5.07%	7.44%	19.33%	5.05%
Cardiac ATDPCs smooth	**83.10%**	**44.31%**	**5.40%**	**16.96%**	69.55%
Subcutaneous ATDPCs vertical	18.72%	11.19%	5.46%	9.32%	8.97%
Subcutaneous ATDPCs horizontal	**70.74%**	**56.72%**	**10.64%**	**26.37%**	15.41%
Subcutaneous ATDPCs smooth	39.29%	57.89%	6.64%	7.20%	6.08%

Bold values are the most relevant for each cell type and condition.

Mechanostimulated cardiac ATDPCs grown on vertical surfaces also differentially expressed proteins that regulated the immune response (complement C3 protein), ECM assembly/disassembly (tissue inhibitor of metalloproteinase 1 [TIMP-1], vimentin, and collagen type VI α-1 chain), angiogenesis (fibronectin [FN]), and cell adhesion (FN, collagen type VI α-1 chain).

Mechanostimulated cardiac ATDPCs grown on smooth surfaces also differentially expressed proteins involved in ECM assembly/disassembly (TIMP-1, matrix metalloproteinase 2 [MMP-2], collagen type VI α-1 chain), angiogenesis (MMP-2), immune response (pentraxin-related protein 3 [PTX3]), cardiomyogenesis and proliferation (follistatin-related protein 1 [FSTL1]), and cell adhesion (collagen type VI α-1 chain).

And mechanostimulated subcutaneous ATDPCs seeded on horizontal surfaces expressed proteins for ECM assembly/disassembly (α-2-macroglobulin [α-2-M]), transcription regulation and cell proliferation (Thioredoxin [TXN]), and cell adhesion (collagen type VI α-1 chain).

Based on a topological analysis, effector proteins involved in MI and cardiac regeneration were observed in all secretomes (except cardiac ATDPCs cultured on horizontal surfaces) (Table 3). Both cardiac and subcutaneous ATDPCs expressed relevant MI and/or cardiac regeneration effectors, such as TIMP-1, FSTL1, or FN. In addition, secretome proteins were directly linked to other effector proteins, such as matrix metalloproteinases (MMP-1, MMP-2, MMP-3, MMP-9, MMP-14, MMP-15), growth factors (TGF-β1 [P01137], FGF-2 [P09038], and IGF-1 [P05019]), and chemokines (CXCR4 [P61073], CXCL12 [P48061], CXCL8 [P10145]), among others (Fig. 3C–E).

Immunostainings for some ECM proteins confirmed secretome data (Table 3). Fibronectin protein expression was increased in mechanically stimulated cardiac ATDPCs cultured on vertical surfaces, and vimentin protein expression was increased in mechanically stimulated subcutaneous ATDPCs cultured on smooth surfaces (Fig. 3F,G).

Of note, some proteins were only detected after mechanical stimulation, such as cartilage oligomeric matrix protein (P49747) or glucose-6-phosphate 1-dehydrogenase (P11413).

## Discussion

It is well known that cells respond to mechanical forces by activating specific genes and signaling pathways that allow them to adapt to their physical environment. Here we conceived and developed a novel, custom-designed, magnet-driven, mechanostimulation device that mimics physiological heart contractions and it was tested in *in vitro* and *in silico* environments.

Cardiac ATDPCs are an attractive source for cell therapies; they can be readily obtained, they constitutively express the primary cardiac genes, they have immunomodulatory properties, and they can differentiate to both cardiac and endothelial lineages, as shown when implanted over the infarcted murine myocardium^11,15–18^. Our strategy was to use biophysical stimulation to induce transdifferentiation of cardiac ATDPCs to the cardiovascular lineage. First, we found that 7 days of mechanical stimulation modulated gene expression in cardiac ATDPCs seeded on all 3 surface patterns. Moreover, we found that the surface pattern influenced cardiac gene modulation. Indeed, early transcription factors were upregulated in cardiac ATDPCs grown on vertical and horizontal patterns, and structural genes were increased in ATDPCs grown on horizontal and smooth patterns. The surface pattern can assist with the cell–cell contact, which is essential for a proper cardiac differentiation. Then, after the cell–cell interaction is reached; the differentiation success would also be determined by other factors, such as culture medium or stretching^19^. In the same line, biophysical cues can be sensed and transduced into intracellular responses to regulate downstream gene expression and stem cell fate, a process known as mechanotransduction^20^. In addition, mechanical forces might have the potential to directly regulate the cell fate through modulating calcium signals^21^. On one hand, mechanical stress activates stretch-activated channels, which causes cytoskeletal remodeling and regulates cell proliferation^22^. On the other hand, mechanical stress on cells attached to the ECM induces integrin rearrangement, and the subsequent differentiation response^23^. Wnt and MAPK signaling pathways are the most frequent; however, others have been described to regulate stem cell differentiation, such as TGF-β1, Notch, Smad and Hedgehog signaling pathways^24^. Taken together, mechanical stimuli affect both proliferation and differentiation, although the complete scheme with all molecular pathways involved remains to be elucidated.

Mechanical conditioning enhanced the expression of transcription factors (GATA-4 and Tbx5) and structural genes (cTnI and α-actinin) in cardiac ATDPCs. On the one hand, GATA-4 is one of the earliest cardiac transcription factors expressed, it is involved in heart development and early cardiac differentiation^25^, and it directly interacts with MEF2 to be co-activators in cardiac myogenesis^26^. Additionally, Tbx5 is crucial for proper cardiovascular development, and it synergistically interacts with GATA-4 and Nkx2.5 transcription factors^27^. On the other hand, cTnI and α-actinin are structural proteins located in the sarcomere, which are responsible for cell alignment. cTnI, the inhibitory subunit of the troponin trimeric complex, binds to actin thin myofilaments to secure the actin-tropomyosin complex in place, and it regulates muscle contraction^28^. The cytoskeletal protein α-actinin cross-links actin filaments and play important roles in cytoskeleton organization and muscle contraction^29^.

We found that the mechanically-stimulated ATDPC secretome was associated with both MI and cardiac regeneration. These functions appear at first antagonistic, but ultimately, they may reflect the two faces of Janus. Certainly, most proteins participated in myocardial structure and function, irrespective of whether the conditions were destructive (MI) or constructive (cardiac regeneration). Interestingly, some transcription factors, such as GATA-4, modulate the paracrine role of cells to support their angiomyogenic potential and cardioprotective effects^30^. Additionally, the patterned surface had a key role to influence the secretion of some factors through the mechanotransduction^20^, and it could be beneficial to design the most appropriate scenarios according to the ultimate goal (i.e. regeneration of the myocardium). The most relevant proteins differentially expressed by mechanostimulation are discussed next.

Complement component C3 activation facilitates myocardial preservation and regeneration through the mobilization and activation of cardiac stem/progenitor cells to promote myocardial regeneration after MI^31^.

LV ECM remodelling post-MI involves MMP activity at every step. MMP activity is regulated by α-2-M and TIMPs. MMP-2 is a proteolytic enzyme, especially secreted under hypoxic conditions, that increases the number of migrating progenitor cells, particularly cardiac stem cells, to damaged tissue^32,33^. TIMP-1 inhibits most MMPs, with high affinity for MMP-9, which is released a few hours after a MI^34^. Further, α-2-M is a homotetramer that can inhibit proteinases, but it is also a secreted glycoprotein present in the plasma of patients with heart failure^35,36^. Consequently, in most of the tested conditions, modulation of α-2-M and TIMP-1 would constrain adverse cardiac remodelling and assist cardiac restoration.

Fibronectin (FN) is an ECM protein highly expressed during early development and re-appears after pathological injury. It is required for heart regeneration, because it is involved in tissue repair processes, and it regulates inflammatory cell function, cell proliferation, cell migration, cellular dedifferentiation, fibrosis, and vascularization^37^. In the MI setting, FN is produced by ischaemic cardiomyocytes, particularly from the epicardium, and by local fibroblasts^38^. Vimentin maintains cell integrity, and its increased content in both cardiomyocytes and new small blood vessels wall has been associated to tissue regeneration^39^.

Collagen type VI α-1 chain is an ECM molecule with cytoprotective functions. It counteracts apoptosis and oxidative damage, regulates autophagy and cell differentiation, and even contributes to the maintenance of stemness^40^. Its presence is linked to early remodelling after acute MI, being a marker of collagen denaturation, although it also contributes to ECM formation at the infarcted zone^41^. Thus, collagen type VI α-1 chain is associated with both MI and cardiac regeneration. We found that it was increased in cardiac ATDPCs and decreased in subcutaneous ATDPCs.

PTX3 is highly expressed in the heart, rapidly expressed during primary local activation of innate immunity and inflammation, and it is considered a cardiac prognosis biomarker^42^. Cardiac microenvironment *in vivo* and mechanical stretching *in vitro* may enhance PTX3 secretion from ATDPCs to assist myocardial regeneration, as previously described^43^.

FSTL1 is involved in heart development and plays a cardioprotective role^44,45^. FSTL1 attenuated hypertrophy following a pressure overload^46^, it prevented myocardial injury in MI and ischemia/reperfusion murine or swine models^44,47^, and it modulated vascular remodelling in response to injury^48^. Recently, the application of human FSTL1 protein in an epicardial patch induced cardiomyocyte proliferation and promoted regeneration, improving cell survival and cardiac function in mouse and swine MI models^44^.

In conclusion, mechanical stimulation of cardiac ATDPCs mimics the cardiac structural milieu. This condition enhanced the expression of early and structural cardiac genes *in vitro* and promoted the secretion of cardioprotective factors as evidenced by the secretome analyses. *In silico* analyses of secreted proteins showed that mechanical stimulation of cardiac ATDPCs was highly associated with MI and repair. Taken together, mechanical pre-conditioning on patterned surfaces prior to cell delivery emerges as a promising therapeutic strategy to drive recovery of cardiac function after MI. Therefore, further *in vivo* experimentation with mechanically conditioned cells will be highly recommended to finally unravel the effects derived from the mechanical stimulation.

## Methods

### Custom-designed, magnet-based, mechanical stimulator and cell support device

The design of the cell support system is shown in Fig. 1A (submitted in a patent application: PCT/EP 2012/061224). The chosen polymer was polydimethylsiloxane (PDMS; Sylgard 184, Dow Corning Corp.), which has an elastic modulus in the range of 1.3–3 MPa relative to preparation temperature.

The prototypes of the cell support system were composed of moulds made of poly(methyl methacrylate) (PMMA). The lower parts of both moulds held embedded magnets that helped to maintain the position of the magnets that we embedded in the PDMS pieces during the curing process. The six-piece mould could hold a pattern cast in order to transfer a vertical (perpendicular to the stretching force), or horizontal line pattern (parallel to the stretching force) onto the bottom of the pool section, where cells were seeded. The regular pattern was imprinted into the PDMS using a planed, ruled diffraction grating (1250 grooves/mm; 05RG150-1250-2, Newport) using a high-precision and consistent polyvinylsiloxane (Affinis, Coltène Whaledent), as previously described^15^. The magnets were selected to fit into the structure and to provide the required forces. The selected magnet model embedded into the PDMS cell support was a 6 × 2 × 4 mm nickel-plated neodymium magnet from Supermagnete (model Q-06-04-02-HN). The selected magnet model for the external driving forces (two magnets, one placed next to the culture plate and the other on the moving arm) were 10 × 10 × 5 mm nickel-plated neodymium magnets, also from Supermagnete (model Q-10-10-05-N). Two inner, transverse slots next to the pool were created to hold electrodes for electrical stimulation or simultaneous electromechanical stimulation.

The current prototype comprised up to 6 culture plates that can be placed in a support made with a sandwich structure of laser cut PMMA and printed circuit board (FR7) pieces, which hold the fixed magnets. The 6 driving magnets were mounted in an aluminium bar, which was moved with a linear servomotor (LM 2070-040-11, Faulhaber). The motor was driven by a MCLM-3006-S motor controller (Faulhaber), which was operated through a RS-232 port by a LabView (National Instruments) application. Through the user interface, one could programme the frequency of the mechanical stimulation, its duty cycle, the number of pulses, and the pulse amplitude (magnet excursion).

Initially, we chose a 1-Hz trapezoidal waveform with a 50% duty cycle. The rise and fall times of the waveform were programmed in the motor controller to occur over an interval of 100 ms, which roughly imitated the shape of the pressure cycle in the heart^49^. To assess the cycle shape, we assumed that the central part of the cell support would deform according to Hooke’s law (deformation is proportional to the force applied). Therefore, we used a force transducer (WPI-Fort25) to measure the force by attaching a magnet to the sensing lever (Fig. 1B,B′); this magnet was like the magnets embedded in the cell support.

Additionally, to evaluate the cell monolayer deformation and its uniformity along the structure, images were captured in the relaxed and maximum strain positions at a given force. Lengths were measured with the distance measurement tool in the Matlab (Mathworks) ImgTool utility display.

Microscale strain transfer characterization was used to confirm the transference from the substrate to the cell monolayer according to Simmons *et al*.^50^. Briefly, random cell morphologies were adequate fiducial markers to use conventional image correlation algorithms on the paired-paired images before and after stretching, using PIVLAB^51^.

### Human ATDPC isolation and culture

Human ATDPCs were isolated from cardiac (cardiac ATDPCs) and subcutaneous (subcutaneous ATDPCs) adipose tissues collected from patients undergoing cardiac surgery. Informed consent was obtained from all patients, the study protocol was approved by the local Ethics Committee (Germans Trias i Pujol University Hospital Ethics Committee), and it conformed to the principles of the Declaration of Helsinki. Adipose tissue biopsy samples were harvested and processed as previously described^11,15,16^ (see Suppl Materials for details).

Briefly, 3 × 10^4^ ATDPC cells were seeded into the culture pool of each PDMS construct one day before stimulation. The PDMS culture pools had 3 different surface patterns: perpendicular to the stretching force (vertical pattern), parallel to the stretching force (horizontal pattern), and without a pattern (smooth surface). The mechanostimulation protocol consisted of 10% stretching at 1 Hz for 7 days. This experiment was repeated a minimum of 4 times (with at least 3 replicates each) for the *in vitro* experimentation (gene and protein analyses), and 3 times for secretome analyses. Non-stimulated cells were used as a control group for the mechanical conditioning, and subcutaneous ATDPCs were used as a control group for cardiac ATDPCs. Cells not attached to the surface or with an atypical phenotype or morphology, and also PDMS constructs without the appropriate stiffness parameters, were discarded.

### Quantitative real-time RT-PCR and immunocytofluorescence

Total RNA was isolated from cardiac and subcutaneous ATDPCs with the AllPrep RNA/Protein Kit (Qiagen). cDNA was synthesized using random hexamers (Qiagen) and the iScript^™^ One-Step RT-PCR Kit (BioRad Laboratories), according to the manufacturer’s protocol. cDNA was preamplified with the TaqMan^®^ PreAmp Master Mix Kit (Applied Biosystems); then, the solutions were diluted 1:5 with RNase-free water (see Suppl Material for more details on RT-PCR and immunocytofluorescence).

### Supernatant collection and proteome construction

Cell seeding onto PDMS constructs was performed one day before beginning the 7-day mechanical stimulation. On day 5 of stimulation, growth media was replaced with the equivalent volume of serum-free media, and 48 h later (on day 7), the supernatant containing secreted proteins was collected, frozen, and stored at −80 °C for further analysis. The supernatants were obtained from paired samples (stimulated and non-stimulated cells) seeded in each condition (vertical, horizontal, and smooth patterned surfaces). The secreted proteins were isolated and sequenced (see Suppl Material for more details on proteome obtainment).

Protein peptides were identified with the Proteome Discoverer software suite (v1.4.1.14, Thermo Fisher Scientific) and the Mascot search engine (v2.4, Matrix Science^52^). A sequence comparison analysis was conducted with an in-house-generated database, which contained all proteins that corresponded to the human database (Uniprot, October 2015), plus the most common contaminants, as previously described^53^. We used a precursor ion mass tolerance of 7 ppm at the MS1 level, and we allowed up to 3 miscleavages for trypsin. The fragment ion mass tolerance was set to 0.5 Da. Oxidation of methionine and protein acetylation at the N-terminus were defined as variable modifications. Carbamidomethylation of cysteines was set as a fixed modification.

The identified peptides were filtered with a false detection rate (FDR) of <1%. For protein quantification, the log-top3 method was used^54^. In brief, the average area of the 3 most abundant peptides per protein was normalized to the median of the overall dataset, and this normalized area was used as a measure of protein abundance.

### In silico functional and topological analyses of the secretome

The secretome profile linked to each treatment was defined as the statistically most abundant proteins identified. Each secretome was evaluated from functional and topological points of view, considering its relationship to MI, cardiac regeneration, and its previous association with the secretome of adipose tissue-derived stem cells^12^.

Next, we performed an artificial neural network (ANN) analysis to explore connections involved in MI and cardiac regeneration, both in global terms and in terms of each individual pathway (see Suppl Material for details).

### Statistical analysis

Expression levels of cardiac and subcutaneous ATDPCs genes were evaluated with the relative fold-change method. Comparisons of control and stimulated groups were evaluated in paired samples with the Student’s *T*-test. Statistical differences were determined from a minimum of 4 independent experiments for every surface pattern. Protein quantifications for both cell populations (cardiac and subcutaneous ATDPCs) in each condition (stimulated *vs*. unstimulated and the 3 different patterns) were compared with the Student’s *T*-test.

Proteomic data for each condition was analysed with a pairwise, trimmed, mean normalization method adapted from Robinson and Oshlack^55^. For each pairwise experiment, we compared the differentially expressed proteins from control and stimulated replicates. Additionally, among the proteins differentially expressed in cardiac ATDPCs seeded on horizontal patterned surfaces, we determined the most significant differences in expression with Fisher’s exact test for small sample sizes^56^.

All the results are presented as the mean ± SEM. A *P*-value < 0.05 was considered statistically significant, and a *P*-value < 0.10 was considered a clear tendency. Statistical analyses were performed with SPSS Statistics software (version 21, IBM SPSS Inc.).

## Electronic supplementary material

Supplementary Information
