# Author Correction: Unravelling the effects of mechanical physiological conditioning on cardiac adipose tissue-derived progenitor cells *in vitro* and *in silico*

**DOI:** 10.1038/s41598-018-24808-y

**Published:** 2018-04-27

**Authors:** Aida Llucià-Valldeperas, Ramon Bragós, Carolina Soler-Botija, Santiago Roura, Carolina Gálvez-Montón, Cristina Prat-Vidal, Isaac Perea-Gil, Antoni Bayes-Genis

**Affiliations:** 1 ICREC Research Program, Health Science Research Institute Germans Trias i Pujol, Badalona, Spain; 2 Electronic and Biomedical Instrumentation Group, Departament d’Enginyeria Electrònica, Universitat Politècnica de Catalunya, Barcelona, Spain; 3 Center of Regenerative Medicine in Barcelona, Barcelona, Spain; 4 Cardiology Service, Germans Trias i Pujol University Hospital, Badalona, Spain; 5 Department of Medicine, Universitat Autònoma de Barcelona, Barcelona, Spain; 6 CIBER Cardiovascular, Instituto de Salud Carlos III, Madrid, Spain

Correction to: *Scientific Reports* 10.1038/s41598-017-18799-5, published online 11 January 2018

In the original version of this Article, an additional affiliation for Carolina Soler-Botija, Santiago Roura, Carolina Gálvez-Montón, Cristina Prat-Vidal and Antoni Bayes-Genis was omitted. The correct affiliation is listed below:

CIBER Cardiovascular, Instituto de Salud Carlos III, Madrid, Spain.

This has now been corrected in the HTML and PDF versions of this Article, and in the accompanying Supplementary Information file.

