# Supplementary material for: Unravelling the effects of mechanical physiological conditioning on cardiac adipose tissue-derived progenitor cells in vitro and in silico
Source: Sci Rep. 2018 Jan 11;8:499. doi: 10.1038/s41598-017-18799-5 (PMC5764962; doi:10.1038/s41598-017-18799-5)
Supplement: Supplementary file 1 — Supplementary Information [file 41598_2017_18799_MOESM1_ESM.pdf]

Unravelling the effects of mechanical physiological conditioning on cardiac adipose tissue-derived progenitor cells *in vitro* and *in silico*

Aida Llucià-Valldeperas[1,*], Ramon Bragós[2], Carolina Soler-Botija[1,6], Santiago Roura[1,3,6], Carolina Gálvez-Montón[1,6], Cristina Prat-Vidal[1,6], Isaac Perea-Gil[1], Antoni Bayes-Genis[1,4,5,6*].

Affiliations:

[1] ICREC Research Program, Health Science Research Institute Germans Trias i Pujol, Badalona, Spain.

[2] Electronic and Biomedical Instrumentation Group, Departament d'Enginyeria Electrònica, Universitat Politècnica de Catalunya, Barcelona, Spain.
[3] Center of Regenerative Medicine in Barcelona, Barcelona, Spain.
[4] Cardiology Service, Germans Trias i Pujol University Hospital, Badalona, Spain.
[5] Department of Medicine, Universitat Autònoma de Barcelona, Barcelona, Spain.
[6] CIBER Cardiovascular, Instituto de Salud Carlos III, Madrid, Spain.

Word count: 3,969.
Running title: Mechanical conditioning in cardiac ATDPCs

Address for correspondence:

Dra. Aida Llucià-Valldeperas, PhD.

Dr. Antoni Bayes-Genis, MD, PhD, FESC, FHFA.

ICREC Research Program, Health Science Research Institute Germans Trias i Pujol. Crta. Canyet, Camí de les escoles, s/n, 08916 Badalona (Barcelona, Spain).

Tel. +34 93 497 3743. Fax. +34 93 497 8654.

E-mail: aida.llucia@gmail.com, abayesgenis@gmail.com.
Keywords: biophysical stimulation, cardiac ATDPCs, mechanical stretching, myocardial infarction, secretome.

Supplementary Material

Methods

Human ATDPCs isolation and culture

Informed consent was obtained from all patients, the study protocol was approved by the local Ethics Committee (Germans Trias i Pujol University Hospital Ethics Committee), and it conformed to the principles of the Declaration of Helsinki. Tissues were obtained from a total of 37 patients (cardiac adipose tissue samples) and 6 patients (subcutaneous adipose tissue samples). Cells isolated from each tissue source were pooled and used for experiments. Subcutaneous ATDPCs were used as control cells.

Briefly, samples were rinsed with PBS and cut into small pieces, and visible blood vessels were removed; next, cells were isolated by collagenase II (Gibco) digestion. Adhered cells were grown in α-MEM (Sigma) supplemented with 10% foetal bovine serum (Gibco), 1 mM L-glutamine (Gibco), and 1% penicillin/streptomycin (Gibco), and cultured under standard culture conditions (37 °C and 5% CO_2) (1).

Quantitative real-time RT-PCR

Real-time PCR amplifications were performed with 2.5 µL cDNA in a final volume of 10 µL, containing 5 µL TaqMan 2× Universal PCR Master Mix, 2 µL RNAse-free water, and 0.5 µL FAM-labeled primer/probe (Applied Biosystems), including glyceraldehyde-3-phosphate dehydrogenase (GAPDH) (Hs99999905_m1), T-box transcription factor (Tbx5) (Hs00361155_m1), myocyte-specific enhancer factor 2A (MEF2A) (Hs01050409_m1), GATA-binding protein 4 (GATA-4) (Hs00171403_m1), α-actinin (ACTN1 gene) (Hs00241650_m1), cardiac Troponin I (cTnI, TNNI3 gene) (Hs00165957_m1), connexin43 (Cx43, GJA1 gene) (Hs00748445_s1), sarco/endoplasmic reticulum Ca^{2+}-ATPase (SERCA2, ATP2A2 gene) (Hs00544877_m1), and β-myosin heavy chain 7 (β-MyHC, MYH7 gene) (Hs00165276_m1).

The following cardiac markers were evaluated: transcription factors (Tbx5, MEF2A, GATA-4), structural genes (α-actinin, cTnI, β-MyHC), and calcium-handling related genes (Cx43, SERCA2). Data were collected and examined on the Light Cycler® 480 Real-Time PCR System (Roche); each sample was analysed in duplicate. The Livak method (2) was used to quantify the absolute ($2^{-\Delta\Delta CT}$) and relative ($2^{-\Delta CT}$) expression of each gene between mechanically conditioned and control samples, using GAPDH as an endogenous reference. The ratio of the 2 conditions (control and stimulated) were collected and calculated as the fold expression (stimulated/control).

Immunocytofluorescence

Cells attached to the PDMS construct were fixed with 10% formalin, permeabilized, blocked in 10% normal horse serum (Gibco) for 1h, and incubated for 1h at room temperature with primary antibodies raised against Cx43 (6.4 µg/mL; Sigma), sarcomeric α-actinin (11.5 µg/mL ascites fluid; Sigma), GATA-4 (4 µg/mL; R&D), MEF2A (4 µg/mL; Santa Cruz), ~~and~~ SERCA2 (4 µg/mL; Santa Cruz), fibronectin (12.6 µg/mL; Abcam) and vimentin (4.7 µg/mL; Abcam). Secondary antibodies were conjugated with Cy2, Cy3 and Cy7 (7.5 µg/mL; Jackson ImmunoResearch), and actin fibers (actinF) were stained with Phalloidin Alexa 568 (0.161 µM; Invitrogen). Nuclei were counterstained with DAPI (0.1 µg/mL; Sigma). Images were acquired with the Axio-Observer Z1 ~~inverted~~ microscope (Zeiss). The fluorescence intensity was normalized by DAPI fluorescence and measured with the Zen Blue software (Zeiss) for at least 30 images per condition.

Proteome obtainment

Starting from a volume of 3 mL, secretome samples were concentrated using VivaSpin 15R Centrifugal Filters (3000 MWCO PES, VSO692) up to 250µL, following the manufacturer's recommendations. Concentrated samples were then precipitated with 6 volumes of ice-cold acetone overnight at -20ºC. Precipitated pellets were resuspended in the digestion

buffer (6M Urea, 200mM NH_4HCO_3); followed by consecutive steps of reduction with DL-Dithiothreitol (10 mM, 1 hour at 37 ºC) and alkylation with iodoacetamide (20 mM, 30 min at 25 ºC in darkness). Samples were diluted to 2 M Urea with 200 mM NH_4HCO_3 and digested with endoproteinase Lys-C (WAKO, 1:10 ratio w:w; enzyme:substrate; overnight, 37 ºC) followed by further dilution to 1M Urea with 200 mM NH_4HCO_3 and the addition of sequence-grade trypsin (Promega; 1:10 ratio w:w; enzyme:substrate; overnight, 37 ºC). Peptide mixtures were then acidified with formic acid and desalted using a C18 UltraMicroSpin column (The Nest Group, Inc) (3) prior to LC-MS/MS analysis.

Samples were analyzed in a LTQ-Orbitrap XL mass spectrometer (Thermo Fisher Scientific) coupled to an EasyLC (Proxeon) equipped with a reversed-phase chromatography 12-cm column with an inner diameter of 75 µm, packed with 5 µm C18 particles (Nikkyo Technos Co.). Chromatographic gradients from 93% buffer A, 7% buffer B to 65% buffer A 35% buffer B in 60 min at a flow rate of 300 nL/min, in which buffer A: 0.1% formic acid in water and buffer B: 0.1% formic acid in acetonitrile.

The mass spectrometer was operated in positive ionization mode with nanospray voltage set at 2.5 kV and source temperature at 200 °C. Ultramark 1621 for the FT mass analyzer was used for external calibration prior the analyses. Moreover, an internal calibration was also performed using the background polysiloxane ion signal at m/z 445.1200. The instrument was operated in DDA mode and full MS scans with 1 micro scans at resolution of 60,000 were used over a mass range of m/z 350-1500 with detection in the Orbitrap. Auto gain control (AGC) was set to 1E6, and dynamic exclusion (60 seconds) and charge state filtering disqualifying singly charged peptides were both activated. In each cycle of DDA analysis, following each survey scan the top ten most intense ions with multiple charged ions above a threshold ion count of 5000 were selected for fragmentation at normalized collision energy of 35%. Fragment ion spectra produced via collision-induced dissociation (CID) were acquired in the ion trap, AGC was set to 5E4, isolation window of 2.0 m/z and maximum injection time of 50 ms was used. All data were acquired with Xcalibur software (v2.1.0.1160, Thermo Fisher Scientific).

In silico functional and topological analyses of the secretome

The topological analysis evaluates direct and indirect links between the proteins that define the secretome profile of each treatment and the proteins included in the description of the following processes: myocardial infarction (MI), cardiac regeneration and Gene Ontology (GO) terms (4) associated (myoblast differentiation, cardiac muscle cell proliferation, cardiomyocyte proliferation, cardiomyocyte apoptosis, and cardiomyocyte differentiation).

Then, an Artificial Neural Network (ANN) analysis has been performed against MI and cardiac regeneration, in global terms and each motive individually. ANNs are supervised algorithms which identify relations between protein sets and clinical elements of the network that are used for training a classifier with the information contained in DrugBank about drugs and indications. The accuracy of the ANNs to reproduce the indications of Drugbank is 98% for those drugs with all targets in the human biological network after applying a cross-fold validation process. Specifically, the ANNs identify possible relations between regions of the network providing a predictive value that quantifies the probability of the existence of a relationship between the evaluated regions, based on the validation of the predictive capacity of the models towards what is described in databases.

Protein interaction networks obtained from the ANN were represented through Cytoscape (5) sessions. Red octagons are secretome proteins; red diamonds are secretome proteins that are also effectors of MI or cardiac regeneration; yellow circles are effectors of MI or cardiac regeneration that are linked to the secretome proteins; the blue circle indicates a protein included in the Gene Ontology (GO) terms that was linked to the indicated secretome protein; and green circles indicate proteins or effectors linked to the secretome proteins.

References

1. Bayes-Genis A., et al. Human progenitor cells derived from cardiac adipose tissue ameliorate myocardial infarction in rodents. J. Mol. Cell. Cardiol. **49**(5), 771-80 (2010).
2. Livak K.J., Schmittgen T.D. Analysis of relative gene expression data using real-time quantitative PCR and the 2–ΔΔCt method. Methods. **25**, 402–408 (2001).
3. Rappsilber J., Mann M., Ishihama Y. Protocol for micro-purification, enrichment, pre-fractionation and storage of peptides for proteomics using StageTips. Nat. Protoc. **2**, 1896-906 (2007).
4. Nucl. Acids Res. (28 January 2015) 43 (D1):D1049-1056. http://geneontology.org/
5. Shannon P., et al. Cytoscape: a software environment for integrated models of biomolecular interaction networks. Genome Res. **13**(11), 2498- 504 (2003). http://www.cytoscape.org/

Supplementary Table

Supplementary Table 1. Relative expression of cardiac markers for each surface condition in subcutaneous ATDPC cultures. Gene expressions were analysed in duplicate for comparisons between mechanically stimulated (MS) and control (Con) samples. Relative expression ($2^{-\Delta CT}$) and fold-changes in expression (MS/Con) are shown for cardiomyogenic genes. Values were normalized to GAPDH expression and represent the mean ± SEM for at least 4 independent experiments. *$P < 0.05$ (significant) and #$P < 0.10$ (trend).

Supplementary Table 1. Cardiac markers relative expression for each condition in subcutaneous ATDPCs culture.

	Sample	Tbx5	MEF2A	GATA-4	α-actinin	Cx43	SERCA2	β-MyHC
Vertical	**sub ATDPCs Con**	0.002 ± 0.001	0.045 ± 0.012	0.004 ± 0.003	0.819 ± 0.088	0.454 ± 0.112	0.148 ± 0.037	0.007 ± 0.007
	**sub ATDPCs MS**	0.004 ± 0.001	0.054 ± 0.016	0.020 ± 0.012	0.910 ± 0.189	0.693 ± 0.315	0.163 ± 0.041	0.030 ± 0.029
	**Ratio sub ATDPCs**	**2.053**	**1.204**	**5.678**	**1.111**	**1.528**	**1.098**	**4.167**
	***P*-value Con vs MS**	***0.007**	0.656	0.266	0.655	0.504	0.804	0.471
Horizontal	**sub ATDPCs Con**	0.003 ± 0.001	0.030 ± 0.008	$5.40\cdot10^{-6} \pm 3.60\cdot10^{-6}$	0.518 ± 0.135	0.592 ± 0.187	0.093 ± 0.019	$5.22\cdot10^{-5} \pm 4.31\cdot10^{-5}$
	**sub ATDPCs MS**	0.004 ± 0.001	0.049 ± 0.014	$8.61\cdot10^{-5} \pm 5.72\cdot10^{-5}$	0.812 ± 0.119	0.846 ± 0.341	0.145 ± 0.015	$7.07\cdot10^{-6} \pm 4.19\cdot10^{-6}$
	**Ratio sub ATDPCs**	**1.269**	**1.622**	**15.948**	**1.569**	**1.429**	**1.554**	**3.372**
	***P*-value Con vs MS**	0.390	0.304	0.206	0.154	0.538	**#0.079**	0.239
Smooth	**sub ATDPCs Con**	0.003 ± 0.000	0.030 ± 0.005	0.004 ± 0.004	0.652 ± 0.143	0.487 ± 0.094	0.130 ± 0.023	$7.07\cdot10^{-6} \pm 4.19\cdot10^{-6}$
	**sub ATDPCs MS**	0.004 ± 0.000	0.049 ± 0.011	0.003 ± 0.003	0.710 ± 0.187	0.493 ± 0.095	0.147 ± 0.027	$2.38\cdot10^{-5} \pm 1.78\cdot10^{-5}$
	**Ratio sub ATDPCs**	**1.332**	**1.661**	**0.871**	**1.090**	**1.012**	**1.130**	**1.207**
	***P*-value Con vs MS**	0.379	0.141	0.926	0.808	0.967	0.637	0.737

Gene expressions were analysed in duplicate for comparisons between mechanically stimulated (MS) and control (Con) samples. Relative expression ($2^{-\Delta CT}$) and fold-changes in expression (MS/Con) are shown for cardiomyogenic genes. Values were normalized to GAPDH expression and represent the mean ± SEM for at least 4 independent experiments. *$P < 0.05$ (significant) and #$P < 0.10$ (trend).
